# Crystal structure of unsymmetrical α-di­imine palladium(II) complex *cis*-[{ArN=C(Me)–(Et)C=NAr}PdCl_2_] [Ar = 2,6-(iPr)_2_C_6_H_3_]

**DOI:** 10.1107/S2056989017009616

**Published:** 2017-07-07

**Authors:** Shravan Kumar Ellandula, Cosmos Opoku Amoako, Joel T. Mague, Perumalreddy Chandrasekaran

**Affiliations:** a Department of Chemistry and Biochemistry, Lamar University, Beaumont, TX 77710, USA; b Department of Chemistry, Tulane University, 6400 Freret Street, New Orleans, LA 70118, USA

**Keywords:** crystal structure, α-di­imines, 1,4-di­aza-1,3-butadienes (DAD), palladium(II) complex, polymerization catalyst, unsymmetrical ligand, ligand synthesis

## Abstract

The unsymmetrical α-di­imine ligand *N*-{2-[2,6-bis­(propan-2-yl)phenylimino]pentan-3-yl­idene}-2,6-bis­(propan-2-yl)aniline, [ArN=C(Me)—(Et)C=NAr] [Ar = 2,6-(iPr)_2_C_6_H_3_], (I), and the corresponding palladium complex, *cis*-(*N*-{2-[2,6-bis­(propan-2-yl)phenylimino]pentan-3-yl­idene}-2,6-bis­(propan-2-yl)aniline)di­chlor­ido­palladium(II) 1,2-di­chloro­ethane monosolvate, [PdCl_2_(C_29_H_42_N_2_)]·C_2_H_4_Cl_2_ or *cis*[PdCl_2_{I}], (II), have been synthesized and characterized. The crystal and mol­ecular structure of the palladium(II) complex have been established by single-crystal X-ray diffraction. The compound crystallized along with a 1,2-di­chloro­ethane solvent of crystallization. The coordination plane of the Pd^II^ atom shows a slight tetra­hedral distortion from square-planar, as indicated by the dihedral angle between the PdCl_2_ and PdN_2_ planes of 4.19 (8)°. The chelate ring is folded along the N⋯N vector by 7.1 (1)°.

## Abstract 2

The synthesis and crystal structure of palladium(II) complex, *cis*-[{ArN=C(Me)-(Et)C=NAr}PdCl_2_] (Ar = 2,6-iPr_2_C_6_H_3_), containing unsymmetrical α-di­imine ligand, is reported.

## Chemical context   

α-Di­imines (or) 1,4-di­aza-1,3-butadienes (DAD) are one of the most versatile classes of chelating nitro­gen-donor ligands, and are well known to stabilize several transition metal complexes at various oxidation levels (Bart *et al.*, 2005 ▸; Greene *et al.*, 2014 ▸). Nickel and palladium complexes of α-di­imines are reported to be effective catalysts for various olefin polymerization and co-polymerization reactions (Ittel *et al.*, 2000 ▸). Furthermore, the polymer properties, topology and stability of these catalysts can be tuned by altering the steric and electronic properties of the α-di­imine ligands (Gates *et al.*, 2000 ▸). These observations have motivated the synthesis of several nickel and palladium complexes with α-di­imine ligands containing various substituents at the imine nitro­gen atom (Nakamura *et al.*, 2009 ▸). α-Di­imine ligands may be conveniently prepared by condensation reactions between alkyl or aryl amine with 1,2-diketones. Most of the reported α-di­imine ligands possess molecular *C*2 symmetry, while very few unsymmetrical α-di­imine ligands, obtained by varying the substituents on the nitro­gen atom, have been reported (Jeon & Kim, 2008 ▸). We report herein the synthesis and spectroscopic characterization of the unsymmetrical α-di­imine ligand [ArN=C(Et)—(Me)C=NAr], (I), [Ar = 2,6-i(Pr)_2_C_6_H_3_] and the corresponding palladium complex *cis*-[PdCl_2_{I}] (II), where the α-di­imine ligand backbone contains methyl and ethyl substituents. The crystal structure of compound (II) has been established using single-crystal X-ray diffraction.
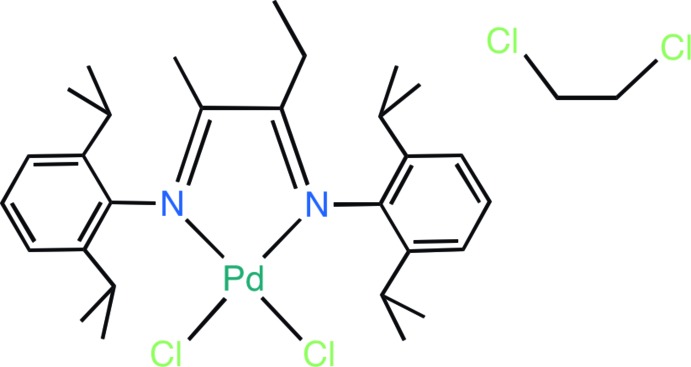


## Structural commentary   

**Figure 1:**
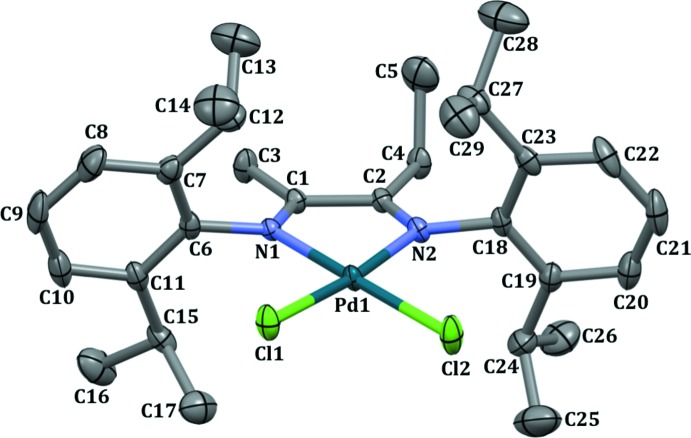
Perspective view of palladium complex (II) with displacement ellipsoids drawn at the 50% probability level. All H atoms and solvent mol­ecule have been omitted for clarity.

The mol­ecular structure of Pd^II^ complex (II), is presented in Fig. 1 ▸. Compound (II) crystallized along with a solvent mol­ecule of 1,2-di­chloro­ethane, which is disordered over the two crystallographic positions. The mol­ecular structure of (II) revels the chelation of the α-di­imine ligand to the palladium(II) atom. The Pd1—N1 and Pd1—N2 distances are 2.0280 (19) and 2.0200 (18) Å, respectively, and are in the typical range for palladium α-di­imine complexes (Zou & Chen, 2016 ▸). The C1—C2 bond length is 1.492 (3) Å, which is slightly shorter than a standard C—C bond length (1.54 Å; Chandrasekaran *et al.*, 2014 ▸), and similarly minimal elongation of the C1—N1 and C2—N2 bonds confirms the slight delocalization of the double bonds. As expected, the palladium(II) atom is in a distorted square-planar geometry, with an N2—Pd1—N1 angle of 79.01 (8)°. The coordination plane shows a slight tetra­hedral distortion from square-planar, as indicated by the dihedral angle between the Cl1–Pd1–Cl2 and N1–Pd1–N2 planes of 4.19 (8)°. The chelate ring is folded along the N1⋯N2 vector by 7.1 (1)°. The aryl substituents at N1 and N2 are nearly perpendicular to the metal–ligand plane, subtending dihedral angles of 81.82 (2)° (C6–C11 aryl ring) and 86.74 (2)° (C18–C23 aryl ring). The aryl substituents in square–planar α-di­imine complexes are anti­cipated to lie perpendicular to the metal–ligand plane due to steric repulsion.

## Supra­molecular features   

**Figure 2:**
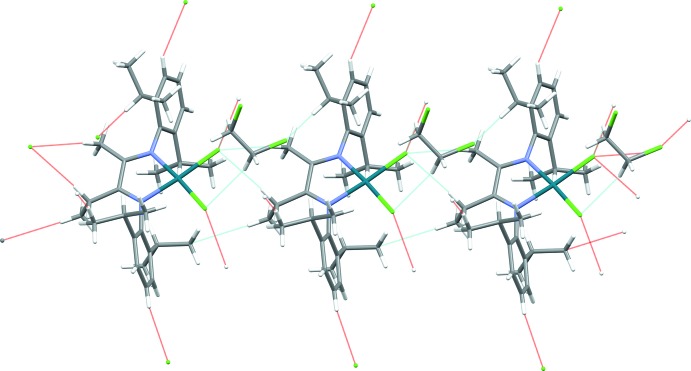
Hydrogen-bonding inter­actions in the crystal lattice.

In the crystal lattice, the components are linked through weak C—H⋯Cl hydrogen-bonding inter­actions between the complex and solvent mol­ecule 1,2-dichloro­ethane (Table 1 ▸, Fig. 2 ▸).

## Database survey   

A search of the Cambridge Structure Database (Version 5.38 with updates Nov 2016; Groom *et al.*, 2016 ▸) confirmed that the Pd^II^ complex *cis*-[{ArN=C(Me)—(Et)C=NAr}PdCl_2_] (Ar = 2,6-(iPr)_2_C_6_H_3_) containing unsymmetrical α-di­imine ligands has not previously been structurally characterized. However, the crystal structures of several Pd^II^ complexes containing symmetrical α-di­imine ligands (IJONIE, Cope-Eatough *et al.*, 2003 ▸; FEGVOD, Coventry *et al.*, 2004 ▸; EBEXAK, Tempel *et al.*, 2000 ▸; APOFOC, Tian *et al.*, 2016 ▸; TABSOH, Chang *et al.*, 2016 ▸) have been reported. In all of these complexes, the Pd^II^ atom exhibits a slightly distorted square-planar geometry.

## Synthesis and crystallization   

**Synthesis of [ArN**=**C(Me)—(Et)C**=**NAr] [Ar = 2,6-(iPr)_2_C_6_H_3_] (I)**. A 100 mL round-bottom flask containing a magnetic bar was charged with 2,3-penta­nedione (1 mL, 0.96 g, 9.6 mmol) and 2,6-diso­propyl­aniline (4.0 mL, 3.76 g, 21.2 mmol). Over this, 50 mL of MeOH was added followed by a few drops of formic acid. The reaction mixture was heated to 343 K for 12 h. It was then cooled to room temperature and the solvent removed under reduced pressure. The resulting yellow pasty solid was dissolved in 15 mL of pentane and stored at 248 K for 3 d, forming a yellow precipitate, which was isolated by filtration and then dried under vacuum, to afford the product as a yellow solid. Yield: 90% (3.63 g). ^1^H NMR (CDCl_3_): 1.08 (*t*, *J* = 7.8 Hz, 3H, CH_2_CH_3_), 1.15 (*d*, *J* = 6.8 Hz, 6H, iPr-CH_3_), 1.20 (*d*, *J* = 6.8 Hz, 6H, iPr-CH_3_), 1.39 (*d*, *J* = 6.7 Hz, 6H, iPr-CH_3_), 1.46 (*d*, *J* = 6.7 Hz, 6H, iPr-CH_3_), 2.05 (*s*, 3H, CH_3_), 2.43 (*q*, *J* = 7.6 Hz, 2H, CH_2_CH_3_), 2.93 (sep, *J* = 6.7 Hz, 2H, iPr-CH), 3.05 (*sep*, *J* = 6.8 Hz, 2H, iPr-CH), 7.08–7.26 (*m*, 6H, Ar-H). IR (cm^−1^): 2957 (*m*), 2926 (*w*), 2868 (*w*), 1631 (*m*), 1458 (*w*), 1433 (*w*), 1362 (*m*), 1323 (*w*), 1254 (*w*), 1183 (*m*), 1123 (*m*), 1056 (*w*), 934 (*w*), 792 (*m*), 761 (*s*), 688 (*w*). Analysis calculated for C_29_H_42_N_2_; C, 83.20; H, 10.11; N, 6.69. Found: C, 83.35; H, 10.07; N, 6.72.

Growing X-ray quality crystals of thw ligand by slow evaporation from various solvents such as hexane, diethyl ether, dicholoro­methane and toluene was unsuccessful.

**Synthesis of *cis*[PdCl_2_{I}] (II)**. A di­chloro­methane (10 mL) solution of [Pd(COD)Cl_2_] (0.10 g, 0.35 mmol) was added dropwise to 5 mL di­chloro­methane solution of (I) (0.15 g, 0.35 mmol) at room temperature. The reaction mixture was stirred for 4 h to give a clear yellow solution. The solvent was removed under reduced pressure, and the resulting yellow solid was washed with 3 × 5 mL of pentane and dried *in vacuo*, affording a yellow powder as the product. Yield: 85% (0.17 g). ^1^H NMR (CDCl_3_): 1.08 (*t*, *J* = 7.8 Hz, 3H, CH_2_CH_3_), 1.20 (*d*, *J* = 6.7 Hz, 12H, iPr-CH_3_), 1.52 (*d*, *J* = 6.7 Hz, 12H, iPr-CH_3_), 2.05 (*s*, 3H, CH_3_), 2.43 (*q*, *J* = 7.8 Hz, CH_2_CH_3_), 2.75 (*sep*, *J* = 6.8 Hz, 2H, iPr-CH), 2.93 (*sep*, *J* = 6.6 Hz, 2H, iPr-CH), 7.08–7.24 (*m*, 6H, Ar-H). IR (cm^−1^): 3031 (*w*), 2989 (*w*), 1523 (*m*), 1478 (*m*), 1448 (*w*), 1419 (*m*), 1341 (*s*), 1310 (*w*), 1247 (*w*), 1177 (*w*), 1088 (*m*), 994 (*s*), 906 (*m*), 865 (*s*), 823 (*m*), 791 (*s*), 767 (*m*). Analysis calculated for C_29_H_42_N_2_PdCl_2_; C, 58.44; H, 7.10; N, 4.70. Found: C, 58.86; H, 7.02; N, 4.94.

X-ray quality crystals of compound (II) were obtained by vapor diffusion of pentane over 1,2-di­chloro­ethane solution.

## Refinement   

Crystal data, data collection and structure refinement details are summarized in Table 2 ▸. H-atoms attached to carbon were placed in calculated positions (C—H = 0.95–1.00 Å). All were included as riding contributions with isotropic displacement parameters 1.2–1.5 times those of the parent atoms. The 1,2-di­chloro­ethane solvent mol­ecule is disordered over two resolved sites in an 0.8596 (15):0.1404 (15) ratio. The minor component was refined with restraints that its geometry approximate that of the major component.

## Supplementary Material

Crystal structure: contains datablock(s) global, I. DOI: 10.1107/S2056989017009616/nk2238sup1.cif

Structure factors: contains datablock(s) I. DOI: 10.1107/S2056989017009616/nk2238Isup2.hkl

CCDC reference: 1559154

Additional supporting information:  crystallographic information; 3D view; checkCIF report

## Figures and Tables

**Table 1:** Hydrogen-bond geometry (Å, °)

*D*—H⋯*A*	*D*—H	H⋯*A*	*D*⋯*A*	*D*—H⋯*A*
C3—H3*B*⋯Cl1^i^	0.98	2.67	3.604 (3)	160
C4—H4*A*⋯Cl1^i^	0.99	2.79	3.763 (3)	166
C15—H15⋯Cl4^i^	1.00	2.80	3.586 (3)	136
C21—H21⋯Cl1^ii^	0.95	2.74	3.633 (3)	156

Symmetry codes: (i) 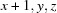; (ii) 
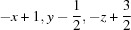
.

**Table 2:** Experimental details

Crystal data	
Chemical formula	[PdCl_2_(C_29_H_42_N_2_)]·C_2_H_4_Cl_2_
*M*_r_	694.90
Crystal system, space group	Monoclinic, *P*2_1_/*c*
Temperature (K)	150
*a*, *b*, *c* (Å)	8.7203 (12), 20.124 (3), 19.526 (3)
β (°)	100.405 (2)
*V* (Å^3^)	3370.2 (8)
*Z*	4
Radiation type	Mo *K*α
μ (mm^−1^)	0.89
Crystal size (mm)	0.12 × 0.07 × 0.06

Data collection	
Diffractometer	Bruker SMART APEX CCD
Absorption correction	Multi-scan (*SADABS*; Bruker, 2013 ▸)
*T*_min_, *T*_max_	0.75, 0.95
No. of measured, independent and observed [*I* > 2σ(*I*)] reflections	61138, 8905, 7064
*R*_int_	0.066
(sin θ/λ)_max_ (Å^−1^)	0.684

Refinement	
*R*[*F* ^2^ > 2σ(*F* ^2^)], *wR*(*F* ^2^), *S*	0.036, 0.084, 1.02
No. of reflections	8905
No. of parameters	366
No. of restraints	3
H-atom treatment	H-atom parameters constrained
Δρ_max_, Δρ_min_ (e Å^−3^)	1.05, −0.72

Computer programs: *APEX2* and *SAINT* (Bruker, 2013 ▸), *SHELXT* (Sheldrick, 2015*a* ▸), *SHELXL2013* (Sheldrick, 2015*b* ▸), *Mercury* (Macrae *et al.*, 2006 ▸) and *SHELXTL* (Sheldrick, 2008 ▸).

## supplementary crystallographic information

### Crystal data

**Table:**

[PdCl_2_(C_29_H_42_N_2_)]·C_2_H_4_Cl_2_	*F*(000) = 1440
*M**_r_* = 694.90	*D*_x_ = 1.370 Mg m^−^^3^
Monoclinic, *P*2_1_/*c*	Mo *K*α radiation, λ = 0.71073 Å
*a* = 8.7203 (12) Å	Cell parameters from 9868 reflections
*b* = 20.124 (3) Å	θ = 2.3–29.0°
*c* = 19.526 (3) Å	µ = 0.89 mm^−^^1^
β = 100.405 (2)°	*T* = 150 K
*V* = 3370.2 (8) Å^3^	Block, orange
*Z* = 4	0.12 × 0.07 × 0.06 mm

### Data collection

**Table:**

Bruker SMART APEX CCD diffractometer	8905 independent reflections
Radiation source: fine-focus sealed tube	7064 reflections with *I* > 2σ(*I*)
Graphite monochromator	*R*_int_ = 0.066
Detector resolution: 8.3660 pixels mm^-1^	θ_max_ = 29.1°, θ_min_ = 2.0°
φ and ω scans	*h* = −11→11
Absorption correction: multi-scan (*SADABS*; Bruker, 2013)	*k* = −27→27
*T*_min_ = 0.75, *T*_max_ = 0.95	*l* = −26→26
61138 measured reflections	

### Refinement

**Table:**

Refinement on *F*^2^	Primary atom site location: structure-invariant direct methods
Least-squares matrix: full	Secondary atom site location: difference Fourier map
*R*[*F*^2^ > 2σ(*F*^2^)] = 0.036	Hydrogen site location: inferred from neighbouring sites
*wR*(*F*^2^) = 0.084	H-atom parameters constrained
*S* = 1.02	*w* = 1/[σ^2^(*F*_o_^2^) + (0.0262*P*)^2^ + 4.3833*P*] where *P* = (*F*_o_^2^ + 2*F*_c_^2^)/3
8905 reflections	(Δ/σ)_max_ = 0.001
366 parameters	Δρ_max_ = 1.05 e Å^−^^3^
3 restraints	Δρ_min_ = −0.72 e Å^−^^3^

### Special details

**Table:**

Experimental. The diffraction data were obtained from 3 sets of 400 frames, each of width 0.5° in ω, collected at φ = 0.00, 90.00 and 180.00° and 2 sets of 800 frames, each of width 0.45° in φ, collected at ω = –30.00 and 210.00°. The scan time was 15 sec/frame.
Geometry. All esds (except the esd in the dihedral angle between two l.s. planes) are estimated using the full covariance matrix. The cell esds are taken into account individually in the estimation of esds in distances, angles and torsion angles; correlations between esds in cell parameters are only used when they are defined by crystal symmetry. An approximate (isotropic) treatment of cell esds is used for estimating esds involving l.s. planes.
Refinement. Refinement of F^2^ against ALL reflections. The weighted R-factor wR and goodness of fit S are based on F^2^, conventional R-factors R are based on F, with F set to zero for negative F^2^. The threshold expression of F^2^ > 2sigma(F^2^) is used only for calculating R-factors(gt) etc. and is not relevant to the choice of reflections for refinement. R-factors based on F^2^ are statistically about twice as large as those based on F, and R- factors based on ALL data will be even larger. H-atoms attached to carbon were placed in calculated positions (C—H = 0.95 - 1.00 Å). All were included as riding contributions with isotropic displacement parameters 1.2 - 1.5 times those of the attached atoms. The dichloroethane solvent molecule is disordered over two resolved sites in an 86:14 ratio. The minor component was refined with restraints that its geometry appoximate that of the major component.

### Fractional atomic coordinates and isotropic or equivalent isotropic displacement parameters (Å^2^)

**Table:**

	*x*	*y*	*z*	*U*_iso_*/*U*_eq_	Occ. (<1)
Pd1	0.58521 (2)	0.59694 (2)	0.75141 (2)	0.01564 (5)	
Cl1	0.37729 (6)	0.66529 (3)	0.75421 (4)	0.02564 (14)	
Cl2	0.41985 (7)	0.51846 (3)	0.69488 (4)	0.03261 (16)	
N1	0.7481 (2)	0.66321 (9)	0.79623 (10)	0.0149 (4)	
N2	0.7819 (2)	0.54249 (9)	0.75778 (10)	0.0161 (4)	
C1	0.8916 (3)	0.64435 (11)	0.79990 (12)	0.0169 (4)	
C2	0.9110 (3)	0.57412 (11)	0.77849 (12)	0.0164 (4)	
C3	1.0301 (3)	0.68623 (12)	0.82792 (15)	0.0255 (5)	
H3A	0.9973	0.7326	0.8309	0.038*	
H3B	1.1064	0.6832	0.7969	0.038*	
H3C	1.0774	0.6704	0.8744	0.038*	
C4	1.0697 (3)	0.54369 (12)	0.78581 (13)	0.0219 (5)	
H4A	1.1408	0.5757	0.7690	0.026*	
H4B	1.0635	0.5036	0.7560	0.026*	
C5	1.1381 (4)	0.52428 (17)	0.86098 (18)	0.0447 (8)	
H5A	1.1389	0.5632	0.8912	0.067*	
H5B	1.2449	0.5081	0.8634	0.067*	
H5C	1.0742	0.4892	0.8764	0.067*	
C6	0.7112 (2)	0.72682 (11)	0.82331 (13)	0.0185 (5)	
C7	0.6592 (3)	0.72574 (13)	0.88714 (14)	0.0244 (5)	
C8	0.6266 (3)	0.78723 (15)	0.91504 (16)	0.0360 (7)	
H8	0.5945	0.7887	0.9590	0.043*	
C9	0.6403 (3)	0.84530 (15)	0.87975 (18)	0.0406 (8)	
H9	0.6177	0.8865	0.8995	0.049*	
C10	0.6868 (3)	0.84447 (13)	0.81571 (17)	0.0341 (7)	
H10	0.6935	0.8851	0.7917	0.041*	
C11	0.7240 (3)	0.78526 (12)	0.78559 (14)	0.0228 (5)	
C12	0.6414 (3)	0.66100 (14)	0.92528 (14)	0.0305 (6)	
H12	0.6311	0.6245	0.8900	0.037*	
C13	0.7856 (4)	0.6460 (2)	0.97959 (19)	0.0546 (9)	
H13A	0.8762	0.6414	0.9567	0.082*	
H13B	0.7698	0.6046	1.0037	0.082*	
H13C	0.8037	0.6825	1.0134	0.082*	
C14	0.4941 (4)	0.6598 (2)	0.95766 (19)	0.0508 (9)	
H14A	0.5043	0.6923	0.9956	0.076*	
H14B	0.4802	0.6153	0.9760	0.076*	
H14C	0.4034	0.6711	0.9221	0.076*	
C15	0.7721 (3)	0.78374 (13)	0.71450 (14)	0.0273 (6)	
H15	0.8556	0.7494	0.7165	0.033*	
C16	0.8396 (4)	0.84954 (16)	0.69451 (19)	0.0442 (8)	
H16A	0.7579	0.8836	0.6881	0.066*	
H16B	0.8794	0.8439	0.6510	0.066*	
H16C	0.9249	0.8634	0.7316	0.066*	
C17	0.6363 (4)	0.76249 (16)	0.65765 (16)	0.0384 (7)	
H17A	0.5981	0.7189	0.6693	0.058*	
H17B	0.6720	0.7598	0.6129	0.058*	
H17C	0.5520	0.7952	0.6544	0.058*	
C18	0.7773 (3)	0.47125 (11)	0.74689 (13)	0.0189 (5)	
C19	0.7886 (3)	0.44508 (12)	0.68145 (14)	0.0237 (5)	
C20	0.7675 (3)	0.37677 (13)	0.67250 (16)	0.0322 (6)	
H20	0.7749	0.3574	0.6289	0.039*	
C21	0.7363 (3)	0.33683 (14)	0.72531 (18)	0.0382 (7)	
H21	0.7187	0.2906	0.7175	0.046*	
C22	0.7304 (3)	0.36388 (13)	0.78997 (17)	0.0338 (7)	
H22	0.7125	0.3354	0.8266	0.041*	
C23	0.7499 (3)	0.43164 (12)	0.80269 (14)	0.0233 (5)	
C24	0.8220 (3)	0.48775 (14)	0.62187 (14)	0.0321 (6)	
H24	0.8367	0.5346	0.6389	0.038*	
C25	0.6858 (4)	0.4866 (2)	0.56041 (18)	0.0575 (10)	
H25A	0.6683	0.4410	0.5433	0.086*	
H25B	0.7103	0.5150	0.5230	0.086*	
H25C	0.5916	0.5032	0.5755	0.086*	
C26	0.9727 (4)	0.46537 (18)	0.59885 (17)	0.0437 (8)	
H26A	1.0602	0.4699	0.6379	0.066*	
H26B	0.9916	0.4931	0.5599	0.066*	
H26C	0.9627	0.4188	0.5840	0.066*	
C27	0.7369 (3)	0.46004 (14)	0.87351 (15)	0.0303 (6)	
H27	0.7809	0.5061	0.8761	0.036*	
C28	0.8298 (5)	0.4201 (2)	0.93406 (19)	0.0575 (10)	
H28A	0.7837	0.3757	0.9352	0.086*	
H28B	0.8267	0.4430	0.9780	0.086*	
H28C	0.9382	0.4159	0.9276	0.086*	
C29	0.5665 (4)	0.46518 (17)	0.88211 (18)	0.0448 (8)	
H29A	0.5082	0.4916	0.8439	0.067*	
H29B	0.5608	0.4867	0.9266	0.067*	
H29C	0.5212	0.4206	0.8813	0.067*	
C30	0.2422 (5)	0.6816 (2)	0.5728 (2)	0.0542 (11)	0.8596 (15)
H30A	0.3380	0.6585	0.5652	0.065*	0.8596 (15)
H30B	0.2716	0.7124	0.6126	0.065*	0.8596 (15)
C31	0.1302 (4)	0.63193 (19)	0.5906 (2)	0.0400 (8)	0.8596 (15)
H31A	0.1851	0.6022	0.6274	0.048*	0.8596 (15)
H31B	0.0916	0.6044	0.5490	0.048*	0.8596 (15)
Cl3	0.1657 (2)	0.72810 (7)	0.49755 (7)	0.0786 (4)	0.8596 (15)
Cl4	−0.03082 (14)	0.66888 (7)	0.61983 (7)	0.0674 (4)	0.8596 (15)
C30A	0.221 (3)	0.6435 (9)	0.5775 (14)	0.0542 (11)	0.1404 (15)
H30C	0.1800	0.6054	0.5478	0.065*	0.1404 (15)
H30D	0.3091	0.6269	0.6127	0.065*	0.1404 (15)
C31A	0.0988 (15)	0.6665 (12)	0.6140 (9)	0.0400 (8)	0.1404 (15)
H31C	0.1251	0.7116	0.6330	0.048*	0.1404 (15)
H31D	0.0927	0.6364	0.6535	0.048*	0.1404 (15)
Cl3A	0.2944 (13)	0.7025 (4)	0.5251 (4)	0.0786 (4)	0.1404 (15)
Cl4A	−0.0833 (8)	0.6687 (4)	0.5576 (4)	0.0674 (4)	0.1404 (15)

### Atomic displacement parameters (Å^2^)

**Table:**

	*U*^11^	*U*^22^	*U*^33^	*U*^12^	*U*^13^	*U*^23^
Pd1	0.01196 (8)	0.01091 (8)	0.02380 (10)	−0.00011 (6)	0.00254 (6)	−0.00023 (7)
Cl1	0.0149 (2)	0.0192 (3)	0.0426 (4)	0.0026 (2)	0.0049 (2)	−0.0047 (3)
Cl2	0.0189 (3)	0.0178 (3)	0.0571 (5)	−0.0020 (2)	−0.0040 (3)	−0.0095 (3)
N1	0.0152 (8)	0.0123 (9)	0.0173 (9)	0.0000 (7)	0.0029 (7)	0.0009 (7)
N2	0.0143 (8)	0.0135 (9)	0.0212 (10)	0.0008 (7)	0.0046 (8)	0.0024 (8)
C1	0.0158 (10)	0.0155 (11)	0.0197 (11)	−0.0001 (8)	0.0038 (9)	0.0003 (9)
C2	0.0173 (10)	0.0144 (11)	0.0183 (11)	0.0005 (8)	0.0051 (9)	0.0013 (9)
C3	0.0163 (11)	0.0200 (12)	0.0392 (15)	−0.0007 (9)	0.0023 (11)	−0.0067 (11)
C4	0.0132 (10)	0.0201 (12)	0.0326 (14)	0.0021 (9)	0.0050 (10)	−0.0045 (10)
C5	0.0336 (16)	0.0442 (19)	0.053 (2)	0.0115 (14)	0.0008 (15)	0.0008 (16)
C6	0.0129 (10)	0.0150 (11)	0.0268 (13)	0.0009 (8)	0.0017 (9)	−0.0033 (9)
C7	0.0209 (11)	0.0260 (13)	0.0254 (13)	0.0028 (10)	0.0015 (10)	−0.0062 (10)
C8	0.0333 (14)	0.0381 (17)	0.0370 (16)	0.0069 (12)	0.0075 (13)	−0.0174 (13)
C9	0.0378 (16)	0.0240 (15)	0.059 (2)	0.0068 (12)	0.0051 (15)	−0.0209 (14)
C10	0.0300 (14)	0.0151 (13)	0.0554 (19)	0.0022 (10)	0.0026 (13)	−0.0025 (12)
C11	0.0172 (11)	0.0149 (11)	0.0347 (14)	0.0003 (9)	0.0003 (10)	0.0010 (10)
C12	0.0357 (14)	0.0342 (15)	0.0235 (13)	0.0018 (12)	0.0101 (12)	−0.0009 (11)
C13	0.049 (2)	0.068 (3)	0.045 (2)	0.0088 (18)	0.0030 (16)	0.0187 (18)
C14	0.0477 (19)	0.064 (2)	0.047 (2)	−0.0008 (17)	0.0233 (17)	0.0024 (17)
C15	0.0246 (12)	0.0206 (13)	0.0367 (15)	0.0009 (10)	0.0061 (11)	0.0094 (11)
C16	0.0363 (16)	0.0362 (18)	0.057 (2)	−0.0141 (13)	−0.0009 (15)	0.0167 (15)
C17	0.0381 (16)	0.0409 (18)	0.0355 (16)	−0.0128 (13)	0.0043 (13)	0.0061 (14)
C18	0.0136 (10)	0.0115 (10)	0.0310 (13)	0.0004 (8)	0.0026 (9)	−0.0005 (9)
C19	0.0219 (11)	0.0187 (12)	0.0298 (14)	0.0000 (9)	0.0026 (10)	−0.0052 (10)
C20	0.0293 (13)	0.0214 (13)	0.0458 (18)	−0.0015 (11)	0.0062 (13)	−0.0126 (12)
C21	0.0302 (14)	0.0146 (13)	0.071 (2)	−0.0030 (11)	0.0135 (15)	−0.0070 (13)
C22	0.0282 (13)	0.0176 (13)	0.059 (2)	0.0010 (10)	0.0180 (13)	0.0119 (13)
C23	0.0165 (11)	0.0199 (12)	0.0344 (14)	0.0043 (9)	0.0069 (10)	0.0068 (10)
C24	0.0440 (16)	0.0282 (15)	0.0230 (13)	−0.0005 (12)	0.0034 (12)	−0.0029 (11)
C25	0.059 (2)	0.074 (3)	0.0342 (18)	0.009 (2)	−0.0053 (17)	0.0033 (18)
C26	0.0471 (18)	0.055 (2)	0.0325 (16)	−0.0054 (16)	0.0156 (15)	−0.0039 (15)
C27	0.0309 (14)	0.0307 (15)	0.0315 (15)	0.0014 (11)	0.0116 (12)	0.0090 (12)
C28	0.057 (2)	0.076 (3)	0.0402 (19)	0.017 (2)	0.0103 (17)	0.0219 (19)
C29	0.0393 (17)	0.047 (2)	0.054 (2)	0.0057 (14)	0.0258 (16)	0.0071 (16)
C30	0.048 (2)	0.069 (3)	0.045 (2)	−0.008 (2)	0.0088 (19)	−0.006 (2)
C31	0.048 (2)	0.034 (2)	0.036 (2)	0.0034 (17)	0.0031 (17)	−0.0031 (16)
Cl3	0.1267 (13)	0.0573 (8)	0.0562 (7)	−0.0177 (8)	0.0283 (8)	0.0119 (6)
Cl4	0.0621 (7)	0.0740 (8)	0.0735 (8)	0.0170 (6)	0.0320 (6)	0.0125 (7)
C30A	0.048 (2)	0.069 (3)	0.045 (2)	−0.008 (2)	0.0088 (19)	−0.006 (2)
C31A	0.048 (2)	0.034 (2)	0.036 (2)	0.0034 (17)	0.0031 (17)	−0.0031 (16)
Cl3A	0.1267 (13)	0.0573 (8)	0.0562 (7)	−0.0177 (8)	0.0283 (8)	0.0119 (6)
Cl4A	0.0621 (7)	0.0740 (8)	0.0735 (8)	0.0170 (6)	0.0320 (6)	0.0125 (7)

### Geometric parameters (Å, º)

**Table:**

Pd1—N2	2.0200 (18)	C17—H17A	0.9800
Pd1—N1	2.0280 (19)	C17—H17B	0.9800
Pd1—Cl2	2.2840 (7)	C17—H17C	0.9800
Pd1—Cl1	2.2843 (6)	C18—C19	1.402 (3)
N1—C1	1.297 (3)	C18—C23	1.405 (3)
N1—C6	1.443 (3)	C19—C20	1.394 (4)
N2—C2	1.293 (3)	C19—C24	1.516 (4)
N2—C18	1.449 (3)	C20—C21	1.373 (4)
C1—C2	1.492 (3)	C20—H20	0.9500
C1—C3	1.493 (3)	C21—C22	1.384 (4)
C2—C4	1.497 (3)	C21—H21	0.9500
C3—H3A	0.9800	C22—C23	1.391 (4)
C3—H3B	0.9800	C22—H22	0.9500
C3—H3C	0.9800	C23—C27	1.519 (4)
C4—C5	1.532 (4)	C24—C25	1.528 (4)
C4—H4A	0.9900	C24—C26	1.531 (4)
C4—H4B	0.9900	C24—H24	1.0000
C5—H5A	0.9800	C25—H25A	0.9800
C5—H5B	0.9800	C25—H25B	0.9800
C5—H5C	0.9800	C25—H25C	0.9800
C6—C7	1.401 (3)	C26—H26A	0.9800
C6—C11	1.403 (3)	C26—H26B	0.9800
C7—C8	1.402 (4)	C26—H26C	0.9800
C7—C12	1.522 (4)	C27—C29	1.529 (4)
C8—C9	1.373 (5)	C27—C28	1.535 (4)
C8—H8	0.9500	C27—H27	1.0000
C9—C10	1.383 (4)	C28—H28A	0.9800
C9—H9	0.9500	C28—H28B	0.9800
C10—C11	1.393 (4)	C28—H28C	0.9800
C10—H10	0.9500	C29—H29A	0.9800
C11—C15	1.521 (4)	C29—H29B	0.9800
C12—C13	1.521 (4)	C29—H29C	0.9800
C12—C14	1.531 (4)	C30—C31	1.482 (6)
C12—H12	1.0000	C30—Cl3	1.768 (5)
C13—H13A	0.9800	C30—H30A	0.9900
C13—H13B	0.9800	C30—H30B	0.9900
C13—H13C	0.9800	C31—Cl4	1.772 (4)
C14—H14A	0.9800	C31—H31A	0.9900
C14—H14B	0.9800	C31—H31B	0.9900
C14—H14C	0.9800	C30A—C31A	1.460 (8)
C15—C16	1.528 (4)	C30A—Cl3A	1.761 (6)
C15—C17	1.530 (4)	C30A—H30C	0.9900
C15—H15	1.0000	C30A—H30D	0.9900
C16—H16A	0.9800	C31A—Cl4A	1.762 (5)
C16—H16B	0.9800	C31A—H31C	0.9900
C16—H16C	0.9800	C31A—H31D	0.9900
			
N2—Pd1—N1	79.01 (8)	C15—C17—H17A	109.5
N2—Pd1—Cl2	96.29 (6)	C15—C17—H17B	109.5
N1—Pd1—Cl2	174.30 (5)	H17A—C17—H17B	109.5
N2—Pd1—Cl1	173.66 (6)	C15—C17—H17C	109.5
N1—Pd1—Cl1	95.21 (5)	H17A—C17—H17C	109.5
Cl2—Pd1—Cl1	89.62 (2)	H17B—C17—H17C	109.5
C1—N1—C6	121.05 (19)	C19—C18—C23	122.9 (2)
C1—N1—Pd1	115.15 (15)	C19—C18—N2	120.0 (2)
C6—N1—Pd1	123.80 (14)	C23—C18—N2	116.9 (2)
C2—N2—C18	122.18 (19)	C20—C19—C18	117.1 (2)
C2—N2—Pd1	115.72 (15)	C20—C19—C24	120.1 (2)
C18—N2—Pd1	121.80 (14)	C18—C19—C24	122.8 (2)
N1—C1—C2	114.7 (2)	C21—C20—C19	121.5 (3)
N1—C1—C3	124.3 (2)	C21—C20—H20	119.2
C2—C1—C3	120.83 (19)	C19—C20—H20	119.2
N2—C2—C1	114.69 (19)	C20—C21—C22	120.0 (3)
N2—C2—C4	124.5 (2)	C20—C21—H21	120.0
C1—C2—C4	120.7 (2)	C22—C21—H21	120.0
C1—C3—H3A	109.5	C21—C22—C23	121.7 (3)
C1—C3—H3B	109.5	C21—C22—H22	119.2
H3A—C3—H3B	109.5	C23—C22—H22	119.2
C1—C3—H3C	109.5	C22—C23—C18	116.8 (3)
H3A—C3—H3C	109.5	C22—C23—C27	120.3 (2)
H3B—C3—H3C	109.5	C18—C23—C27	122.9 (2)
C2—C4—C5	112.9 (2)	C19—C24—C25	111.4 (3)
C2—C4—H4A	109.0	C19—C24—C26	110.6 (2)
C5—C4—H4A	109.0	C25—C24—C26	110.6 (3)
C2—C4—H4B	109.0	C19—C24—H24	108.0
C5—C4—H4B	109.0	C25—C24—H24	108.0
H4A—C4—H4B	107.8	C26—C24—H24	108.0
C4—C5—H5A	109.5	C24—C25—H25A	109.5
C4—C5—H5B	109.5	C24—C25—H25B	109.5
H5A—C5—H5B	109.5	H25A—C25—H25B	109.5
C4—C5—H5C	109.5	C24—C25—H25C	109.5
H5A—C5—H5C	109.5	H25A—C25—H25C	109.5
H5B—C5—H5C	109.5	H25B—C25—H25C	109.5
C7—C6—C11	123.3 (2)	C24—C26—H26A	109.5
C7—C6—N1	116.2 (2)	C24—C26—H26B	109.5
C11—C6—N1	120.5 (2)	H26A—C26—H26B	109.5
C6—C7—C8	116.9 (3)	C24—C26—H26C	109.5
C6—C7—C12	121.8 (2)	H26A—C26—H26C	109.5
C8—C7—C12	121.3 (2)	H26B—C26—H26C	109.5
C9—C8—C7	120.9 (3)	C23—C27—C29	111.0 (2)
C9—C8—H8	119.5	C23—C27—C28	112.8 (3)
C7—C8—H8	119.5	C29—C27—C28	109.8 (2)
C8—C9—C10	120.7 (3)	C23—C27—H27	107.6
C8—C9—H9	119.7	C29—C27—H27	107.6
C10—C9—H9	119.7	C28—C27—H27	107.6
C9—C10—C11	121.4 (3)	C27—C28—H28A	109.5
C9—C10—H10	119.3	C27—C28—H28B	109.5
C11—C10—H10	119.3	H28A—C28—H28B	109.5
C10—C11—C6	116.7 (2)	C27—C28—H28C	109.5
C10—C11—C15	121.7 (2)	H28A—C28—H28C	109.5
C6—C11—C15	121.6 (2)	H28B—C28—H28C	109.5
C13—C12—C7	111.5 (3)	C27—C29—H29A	109.5
C13—C12—C14	111.0 (3)	C27—C29—H29B	109.5
C7—C12—C14	112.5 (2)	H29A—C29—H29B	109.5
C13—C12—H12	107.2	C27—C29—H29C	109.5
C7—C12—H12	107.2	H29A—C29—H29C	109.5
C14—C12—H12	107.2	H29B—C29—H29C	109.5
C12—C13—H13A	109.5	C31—C30—Cl3	112.7 (3)
C12—C13—H13B	109.5	C31—C30—H30A	109.1
H13A—C13—H13B	109.5	Cl3—C30—H30A	109.1
C12—C13—H13C	109.5	C31—C30—H30B	109.1
H13A—C13—H13C	109.5	Cl3—C30—H30B	109.1
H13B—C13—H13C	109.5	H30A—C30—H30B	107.8
C12—C14—H14A	109.5	C30—C31—Cl4	112.7 (3)
C12—C14—H14B	109.5	C30—C31—H31A	109.0
H14A—C14—H14B	109.5	Cl4—C31—H31A	109.0
C12—C14—H14C	109.5	C30—C31—H31B	109.0
H14A—C14—H14C	109.5	Cl4—C31—H31B	109.0
H14B—C14—H14C	109.5	H31A—C31—H31B	107.8
C11—C15—C16	113.5 (2)	C31A—C30A—Cl3A	116.3 (16)
C11—C15—C17	111.3 (2)	C31A—C30A—H30C	108.2
C16—C15—C17	109.9 (2)	Cl3A—C30A—H30C	108.2
C11—C15—H15	107.3	C31A—C30A—H30D	108.2
C16—C15—H15	107.3	Cl3A—C30A—H30D	108.2
C17—C15—H15	107.3	H30C—C30A—H30D	107.4
C15—C16—H16A	109.5	C30A—C31A—Cl4A	111.0 (17)
C15—C16—H16B	109.5	C30A—C31A—H31C	109.4
H16A—C16—H16B	109.5	Cl4A—C31A—H31C	109.4
C15—C16—H16C	109.5	C30A—C31A—H31D	109.4
H16A—C16—H16C	109.5	Cl4A—C31A—H31D	109.4
H16B—C16—H16C	109.5	H31C—C31A—H31D	108.0

### Hydrogen-bond geometry (Å, º)

**Table:**

*D*—H···*A*	*D*—H	H···*A*	*D*···*A*	*D*—H···*A*
C3—H3*B*···Cl1^i^	0.98	2.67	3.604 (3)	160
C4—H4*A*···Cl1^i^	0.99	2.79	3.763 (3)	166
C15—H15···Cl4^i^	1.00	2.80	3.586 (3)	136
C21—H21···Cl1^ii^	0.95	2.74	3.633 (3)	156

Symmetry codes: (i) *x*+1, *y*, *z*; (ii) −*x*+1, *y*−1/2, −*z*+3/2.
